# Supplementary material for: Discovery and genomics of H2-oxidizing/O2-reducing Deferribacterota ectosymbiotic with protists in the guts of termites and a Cryptocercus cockroach
Source: ISME Commun. 2026 Feb 4;6(1):ycag002. doi: 10.1093/ismeco/ycag002 (PMC12887300; doi:10.1093/ismeco/ycag002)
Supplement: renamed_0cbfb_ycag002 [file renamed_0cbfb_ycag002.pdf]

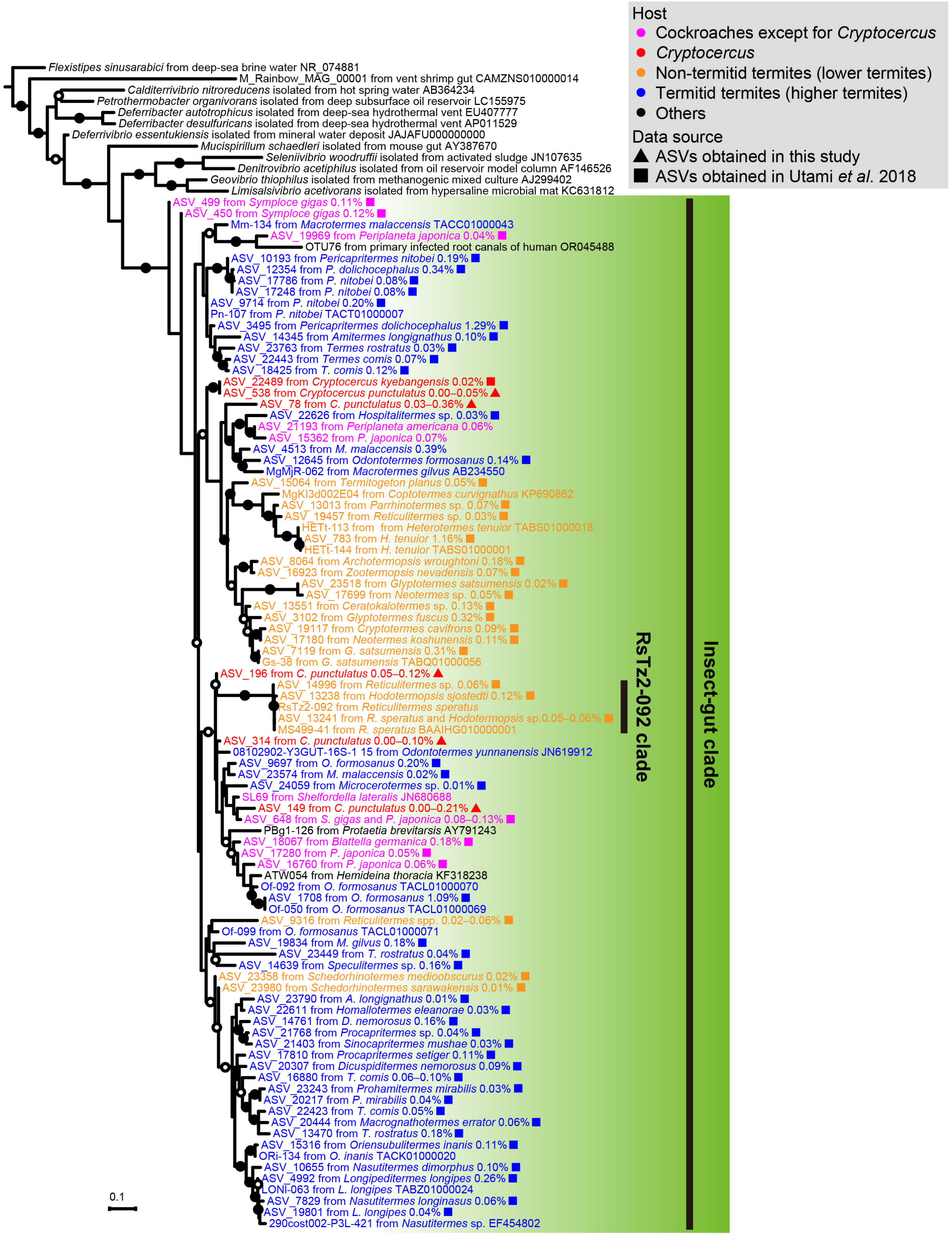

Figure S1. Phylogenetic diversity of *Deferribacterota* in termite and cockroach guts based on the 16S rRNA gene V3–V4 region. A maximum-likelihood tree was constructed using 409 nucleotide positions with the TIM3e+I+G4 substitution model and an outgroup (AB729138). Filled and open circles indicate nodes with ultrafast bootstrap value ≥95% and ≥80%, respectively.

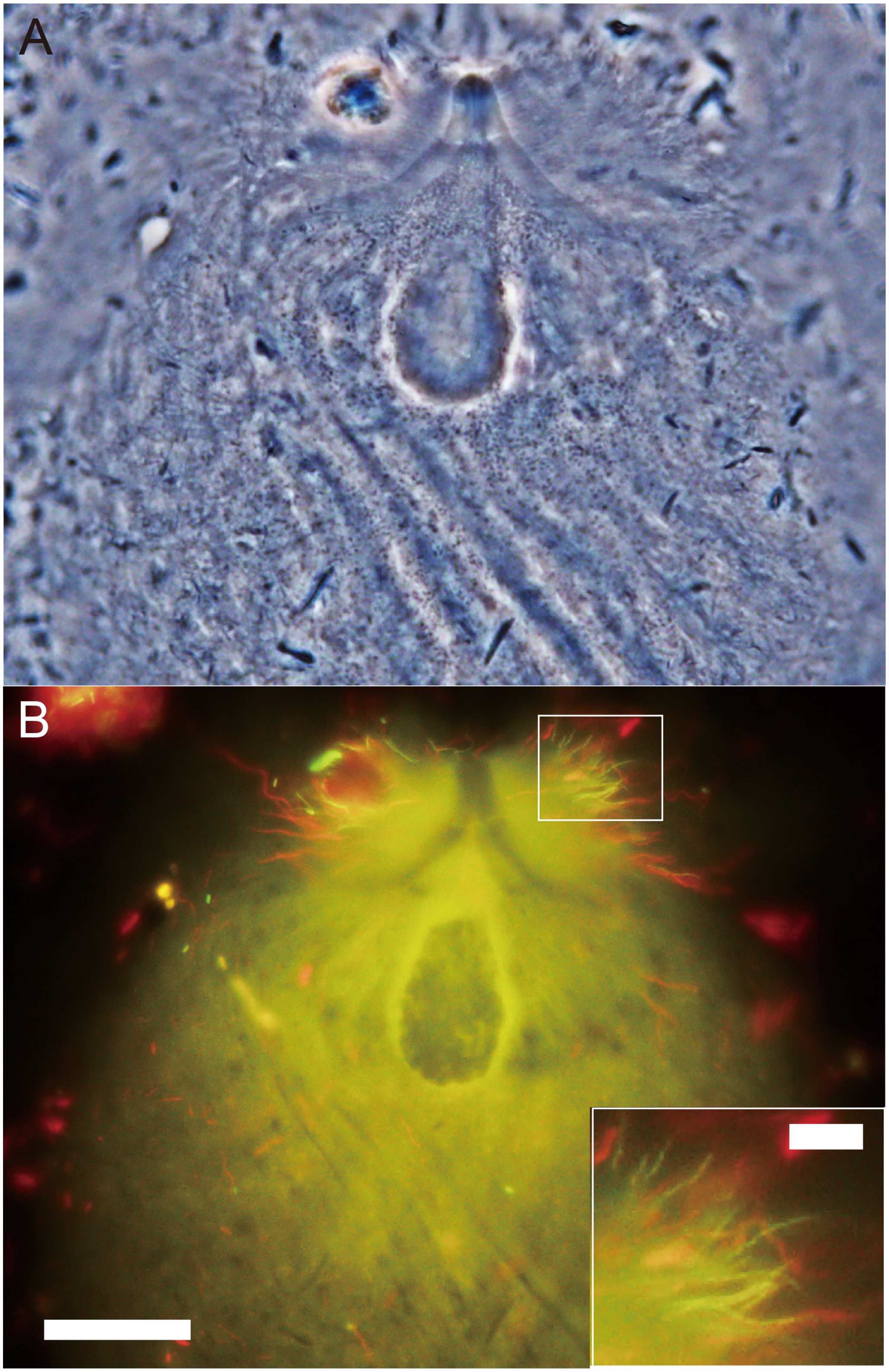

Figure S2. Detection of *Deferribacterota* associated with protists in the gut of *Cryptocercus punctulatus*. (A) Phase-contrast image of *Trichonympha acuta*. (B) Epifluorescence image. FISH using probes Deferri-term-661 labeled with 6FAM (green) and EUB338 labeled with Texas red (red) was performed. The FISH images were overlayed; spiral, rod-shaped cells of *Deferribacterota* were detected with a merged, yellowish color. Magnified image is shown in inset. Bar indicates 50 µm and 10 µm (inset).

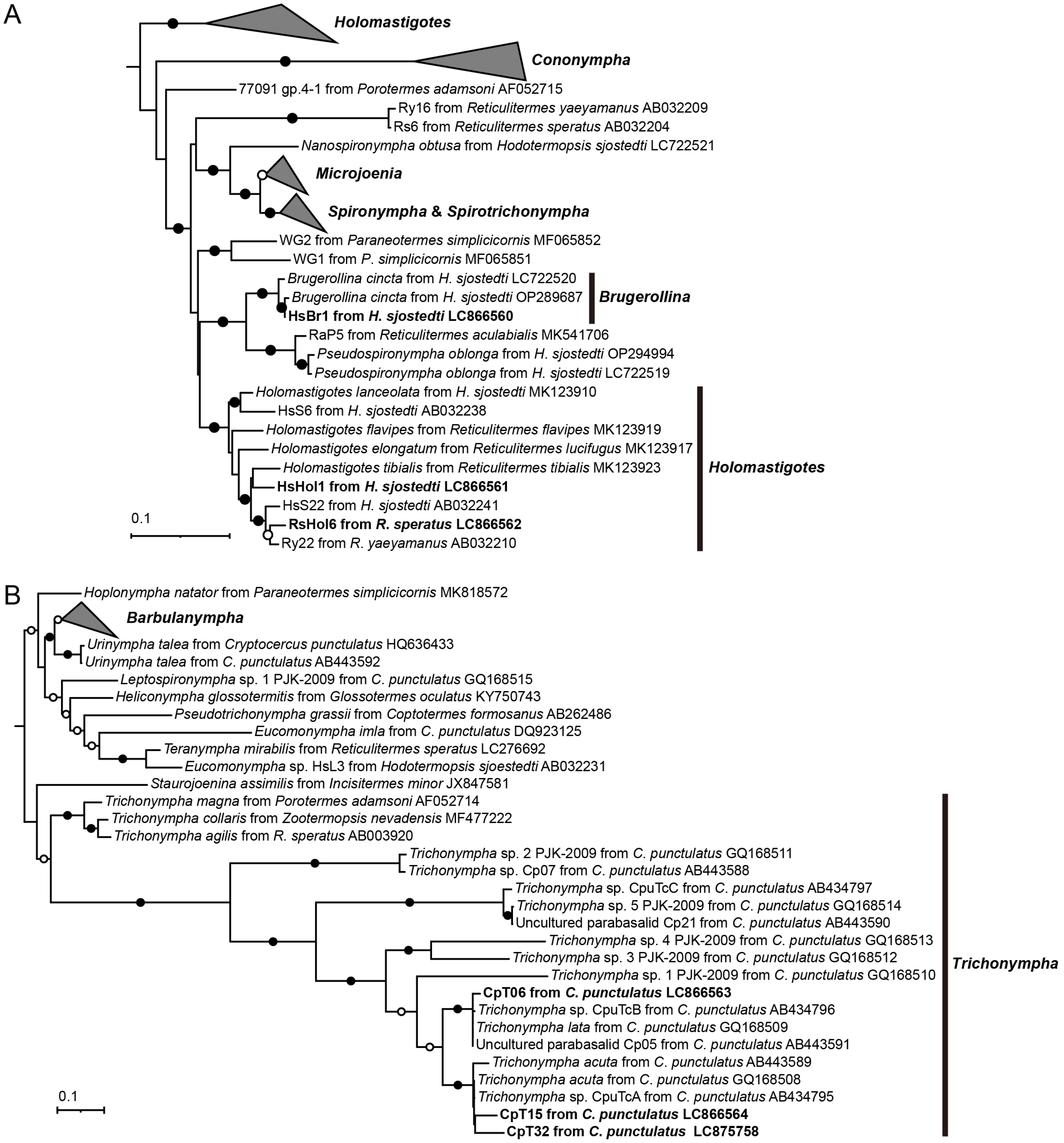

Figure S3. Phylogenetic positions of host protists based on the 18S rRNA gene. Maximum likelihood trees of (A) *Spirotrichonymphida* constructed using 1,364 nucleotide sites with the TVMe+I+G4 substitution model and an outgroup (AY055799, AY319278, GQ254638, and GQ254637) and (B) *Trichonymphida* constructed using 1,276 nucleotide sites with the GTR+F+I+G4 substitution model and an outgroup (AY338476 and JN619423) are shown. The sequences obtained in this study are shown in bold. Filled and open circles indicate ultrafast bootstrap value ≥ 95% and ≥80%, respectively.

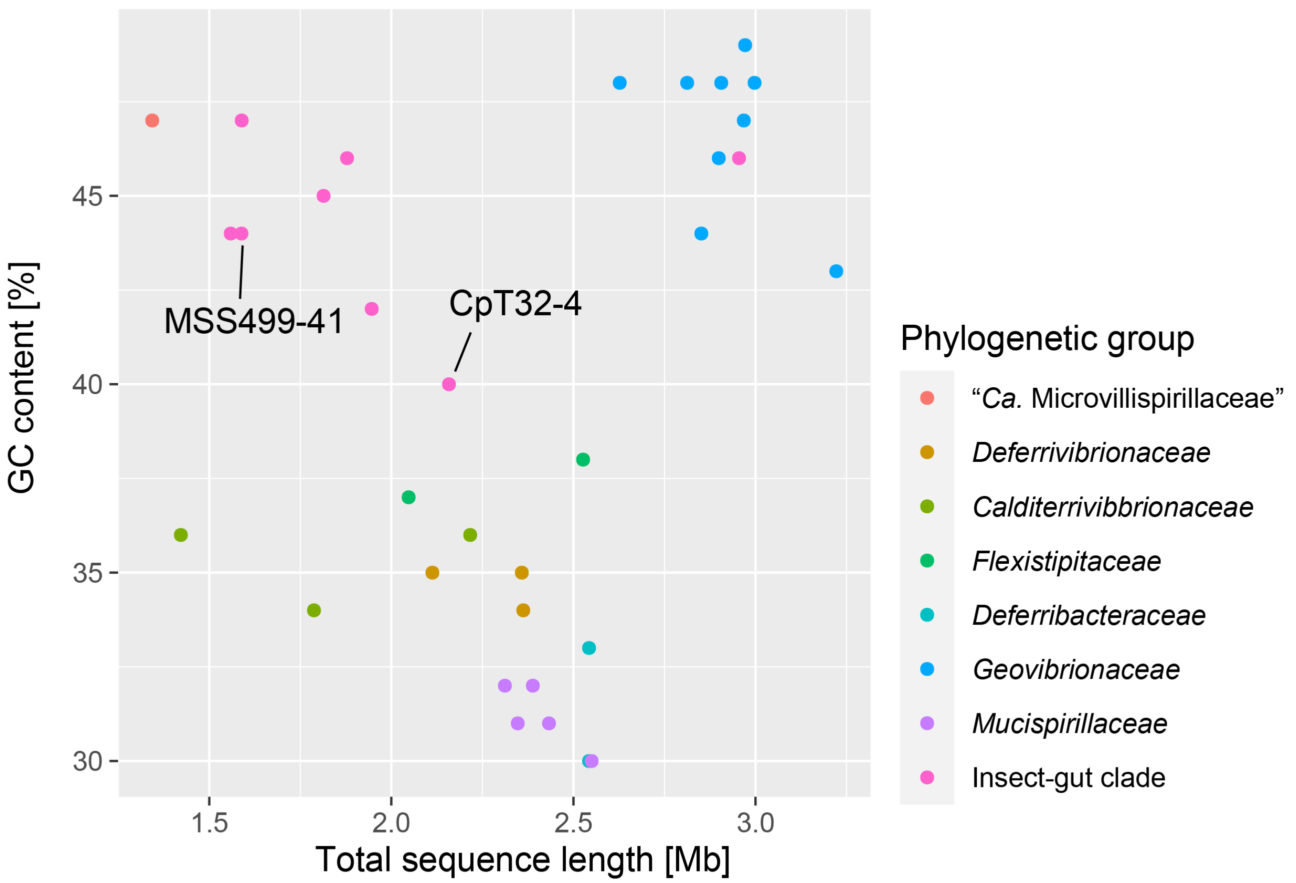

Figure S4. Comparisons of total sequence length and GC content of *Deferribacterota* genomes, which were used in the phylogenomic analysis in Fig. 2B. Genomes with an estimated completeness of <75% were excluded. The genomes of *Termitispirillum cryptocerci* (CpT32-4) and "*Candidatus* Termitispirillum reticulitermitis" (MS499-41) are indicated.

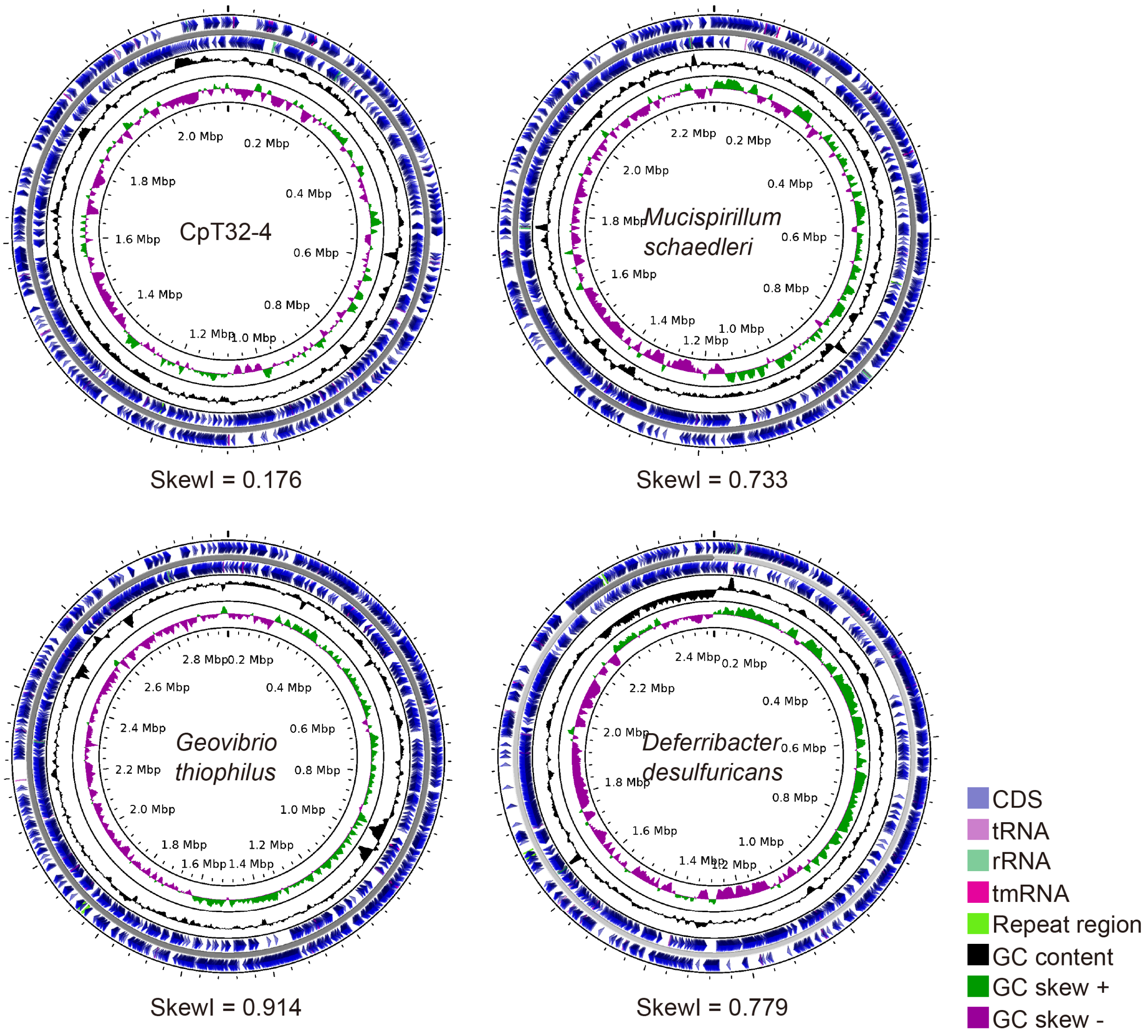

Figure S5. Circular maps of complete chromosomal genomes of four *Deferribacterota* species, including *Termitispirillum cryptocerci* (CpT32-4). Position 1 is set at the initial nucleotide site of the *dnaA* gene. SkewI values are shown.

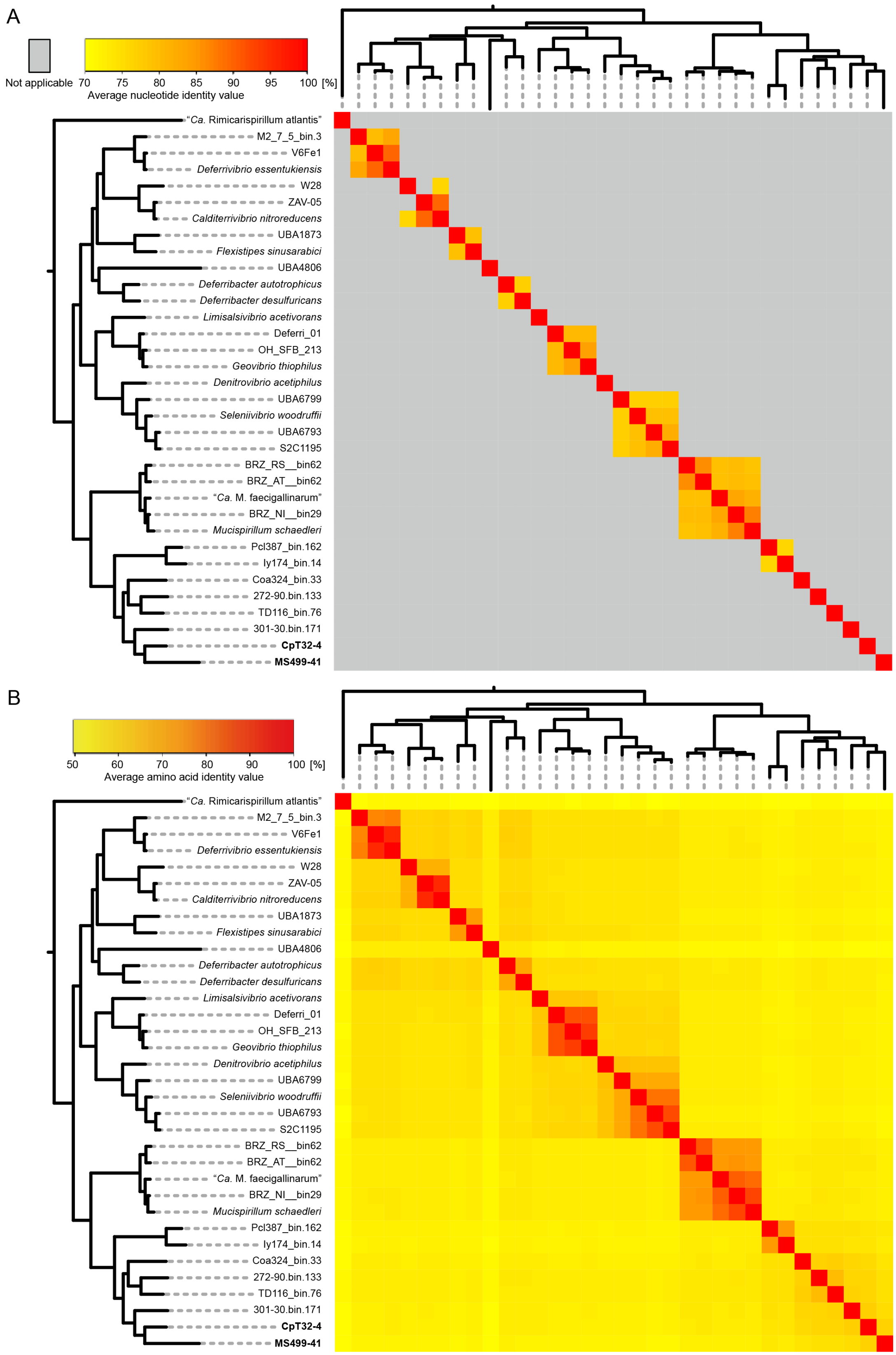

Figure S6. Pairwise sequence identities between *Deferribacterota* genomes, including *Termitispirillum cryptocerci* (CpT32-4) and "*Candidatus* Termitispirillum reticulitermitis" (MS499-41). (A) Average nucleotide identities and (B) average amino acid identities are presented as heatmaps.

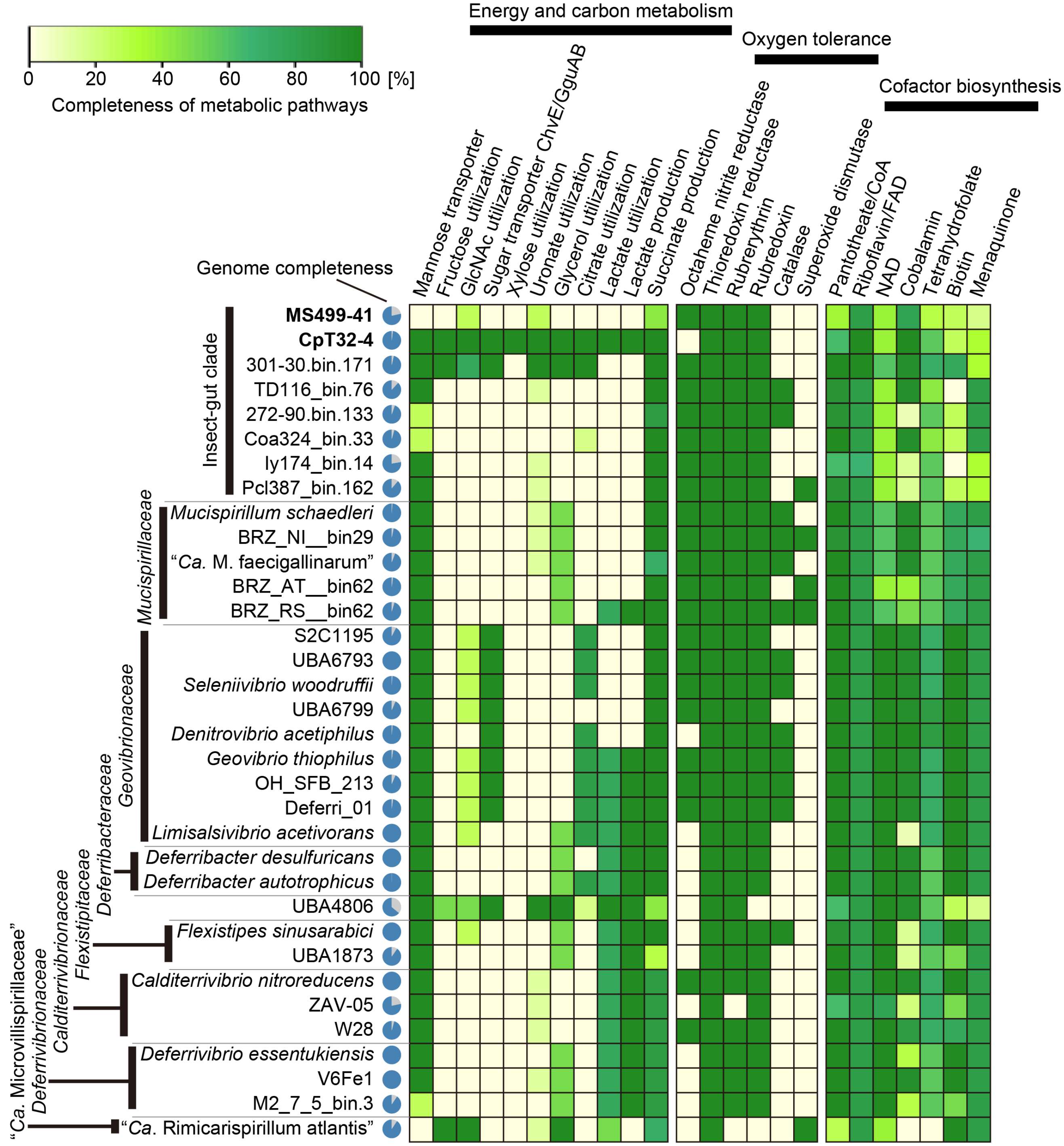

Figure S7. Presence or absence of genes or completeness of metabolic pathways in *Deferribacterota*, including *Termitispirillum cryptocerci* (CpT32-4) and "*Candidatus* Termitispirillum reticulitermitis" (MS499-41).

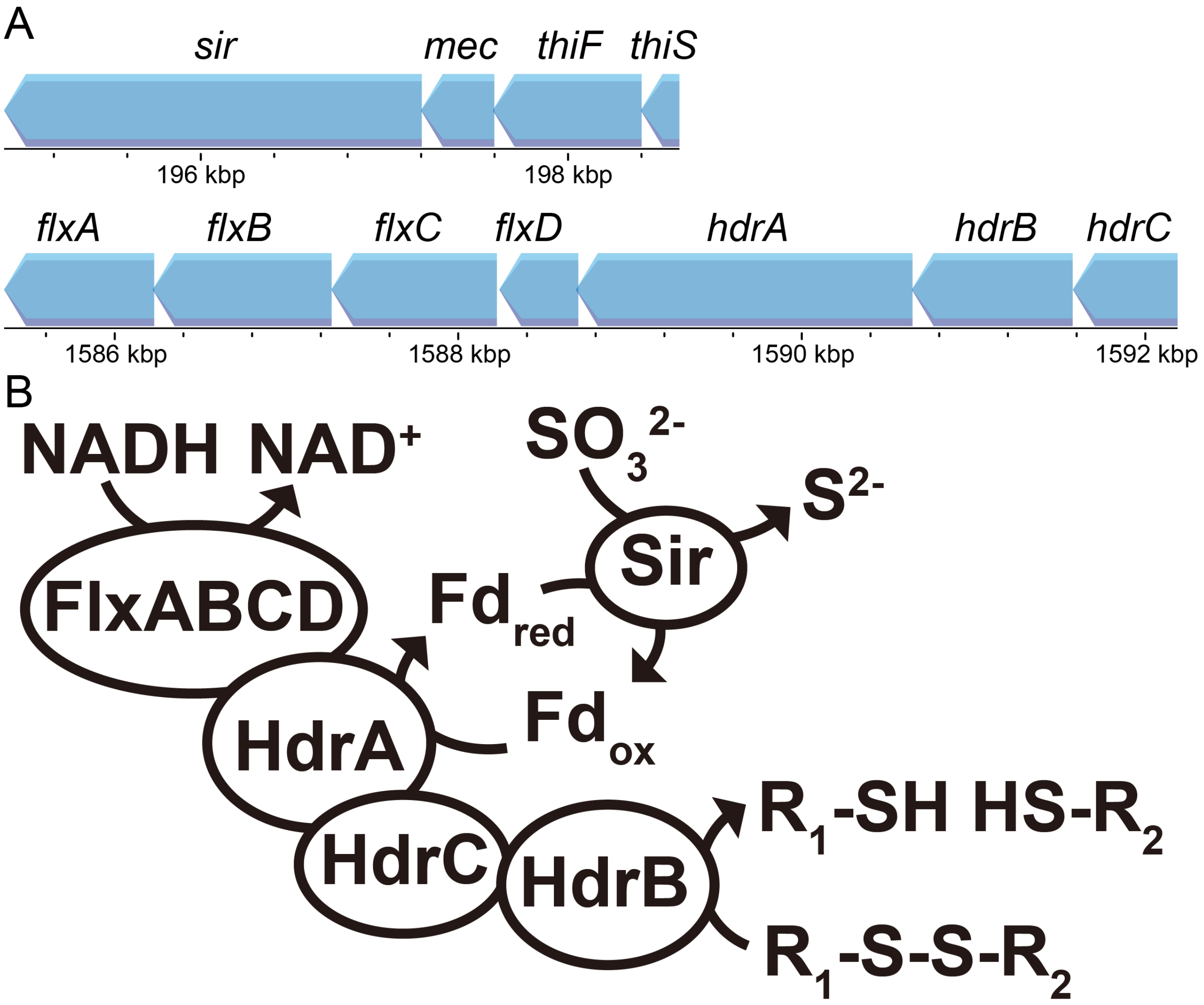

Figure S8. Genes putatively involved in sulfite reduction in *Termitispirillum cryptocerci* (CpT32-4). (A) Loci of genes encoding sulfite reductase (*sir*), [CysO sulfur-carrier protein]-S-L-cysteine hydrolase (*mec*), sulfur carrier protein ThiS adenylyltransferase (*thiF*), sulfur carrier protein (*thiS*), NADH dehydrogenase complex (*flxABCD*), and heterodisulfide reductase complex (*hdrABC*). Arrows indicate the direction of transcription. (B) Predicted scheme of sulfite reduction driven by a flavin-based electron bifurcation.

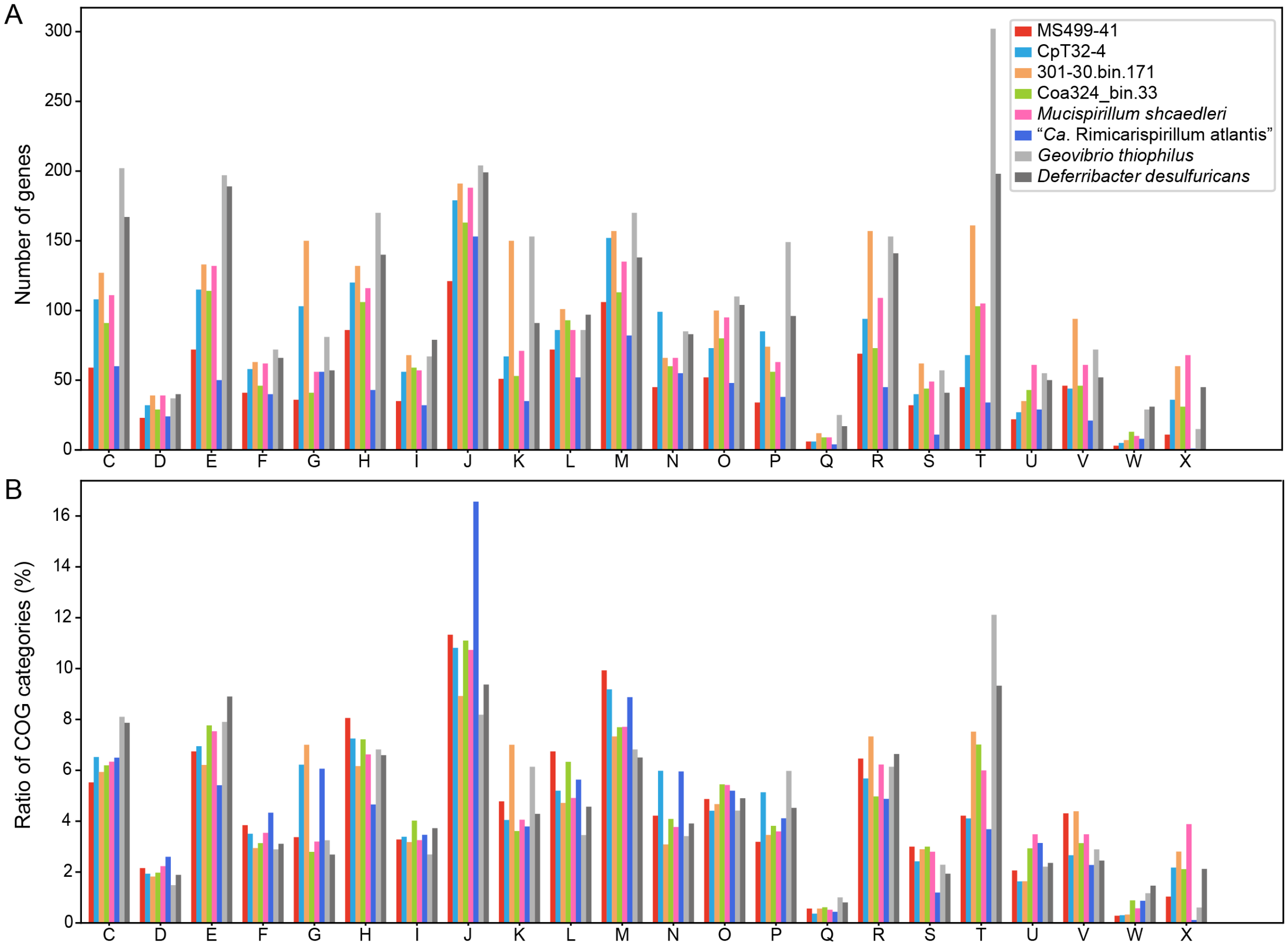

Figure S9. Comparation of genomic contents based on the clusters of orthologous genes (COGs) among representative species of *Deferribacterota*, including *Termitispirillum cryptocerci* (CpT32-4) and "*Candidatus* Termitispirillum reticulitermitis" (MS499-41. (A) Total number and (B) ratio of genes per COG category in each *Deferribacterota* species. The COG categories analyzed are: [C] Energy production and conversion, [D] Cell cycle control, cell division, chromosome partitioning, [E] Amino acid transport and metabolism, [F] Nucleotide transport and metabolism, [G] Carbohydrate transport and metabolism, [H] Coenzyme transport and metabolism, [I] Lipid transport and metabolism, [J] Translation, ribosomal structure and biogenesis, [K] Transcription, [L] Replication, recombination and repair, [M] Cell wall/membrane/envelope biogenesis, [N] Cell motility, [O] Posttranslational modification, protein turnover, chaperones, [P] Inorganic ion transport and metabolism, [Q] Secondary metabolites biosynthesis, transport and catabolism, [R] General function prediction only, [S] Function unknown, [T] Signal transduction mechanisms, [U] Intracellular trafficking, secretion, and vesicular transport, [V] Defense mechanisms, [W] Extracellular structures, [X] Mobilome: prophages, transposons.

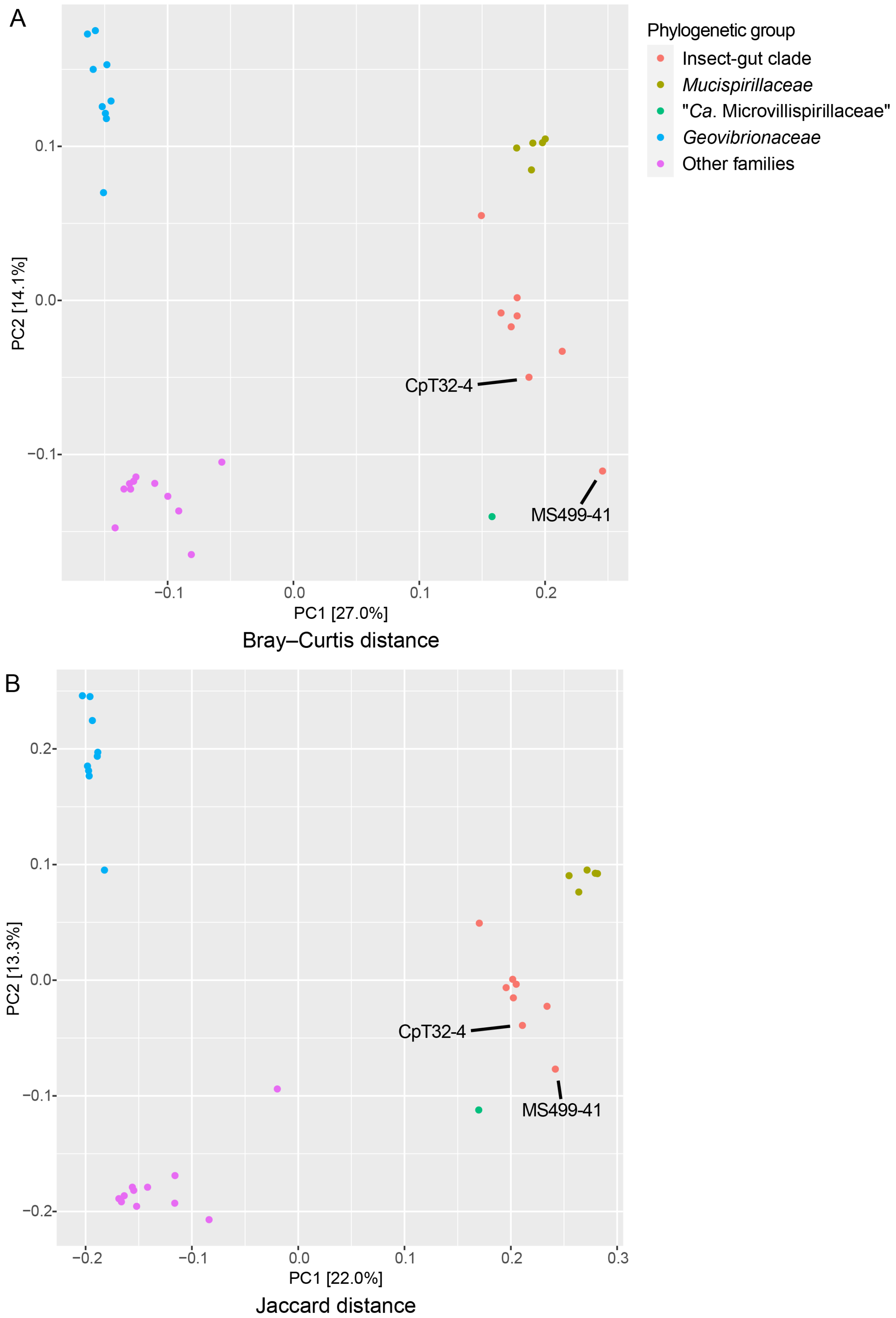

Figure S10. Similarity of genomic content among *Deferribacterota*. PCoA plots based on (A) Bray-Curtis distance and (B) Jaccard distance, calculated from orthogroup compositions. *Termitispirillum cryptocerci* (CpT32-4) and "*Candidatus* Termitispirillum reticulitermitis" (MS499-41) are indicated.

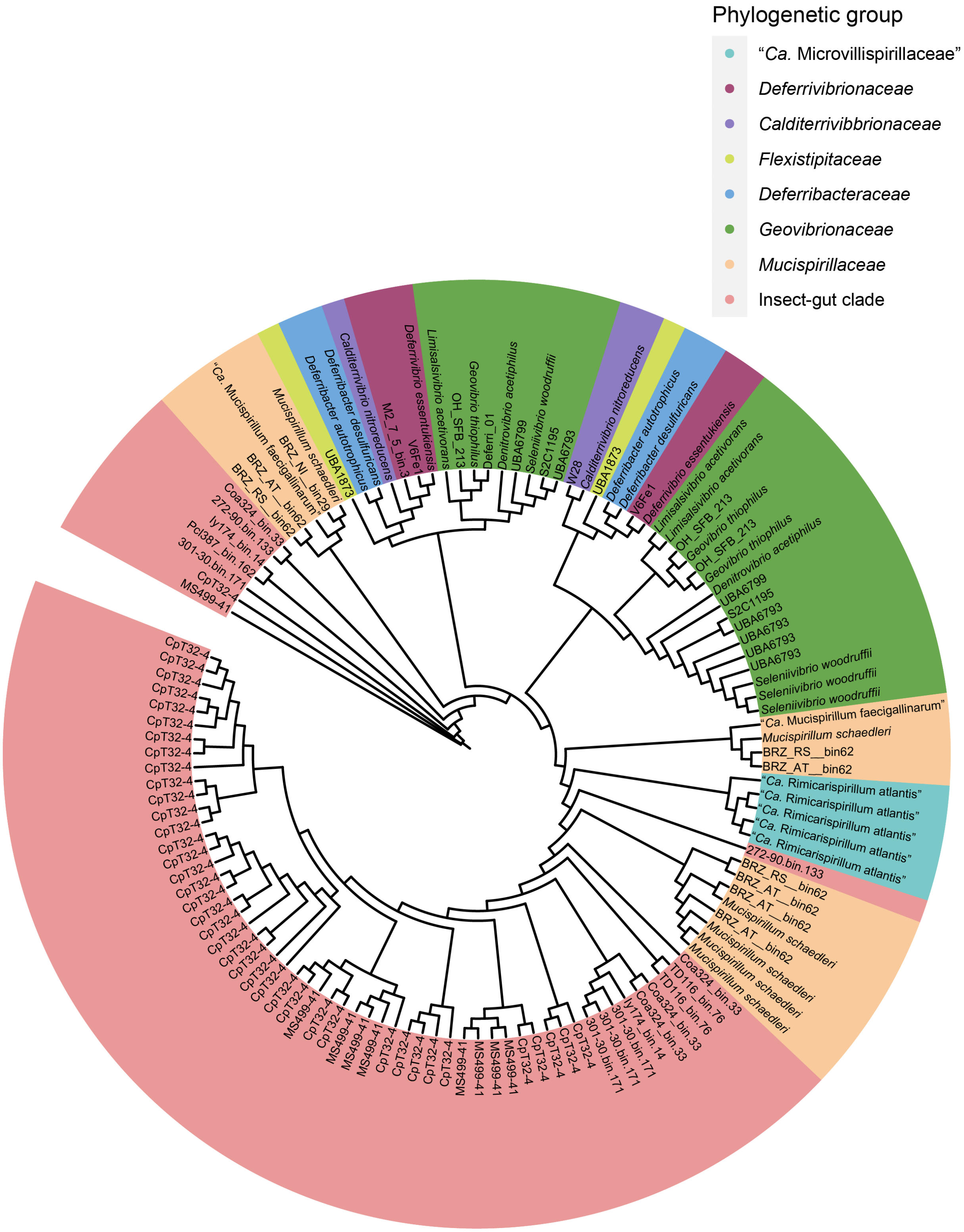

Figure S11. Phylogenetic diversity of the flagellin gene of *Deferribacterota*, including *Termitispirillum cryptocerci* (CpT32-4) and "*Candidatus* Termitispirillum reticulitermitis" (MS499-41). A maximum likelihood tree was constructed with the LG+F+G4 substitution model, based on 624 amino acid positions. Only genomes with the completeness >70% and contamination rate <5% were used as the sources of the flagellin genes.
