# Supplementary material for: Discovery and genomics of H2-oxidizing/O2-reducing Deferribacterota ectosymbiotic with protists in the guts of termites and a Cryptocercus cockroach
Source: ISME Commun. 2026 Feb 4;6(1):ycag002. doi: 10.1093/ismeco/ycag002 (PMC12887300; doi:10.1093/ismeco/ycag002)
Supplement: renamed_a723b_ycag002 [file renamed_a723b_ycag002.pdf]

Table S1. PCR primers used in this study.

Primer name	Target gene	Primer sequence (5′→3′)	Reference
Pro341F	Prokaryote 16S rRNA gene (V3–V4 region)	CCTACGGGNBGCASCAG	[1]
Pro805R	Prokaryote 16S rRNA gene (V3–V4 region)	GACTACNVGGGTATCTAATCC	[1]
27Fmix	*Bacteria* 16S rRNA gene (near full-length)	AGRGTTTGATYMTGGCTCAG	[2]
1492Rmix	*Bacteria* 16S rRNA gene (near full-length)	GGHTACCTTGTTACGACTT	[2]
SpiroF1	*Parabasalia* 18S rRNA gene (outer)	ATACTTGGTCGATCCTGCCAAGG	[3]
SpiroR1	*Parabasalia* 18S rRNA gene (outer)	TGATCCAACGGCAGGTTCMCCTAC	[3]
GGF	*Parabasalia* 18S rRNA gene (inner)	CTTCGGTCATAGATTAAGCCATGC	[4]
GGR	*Parabasalia* 18S rRNA gene (inner)	CCTTGTTACGACTTCTCCTTCCTC	[4]
E23F3	*Eukarya* 18S rRNA gene (near full-length)	ACYTGGTTGATYCTGCC	[5]
E1511R4	*Eukarya* 18S rRNA gene (near full-length)	CWDCBGCAGGTTCWCCWAC	[5]

Table S2. FISH probes used in this study.

Probe name	Target taxon	Probe sequence (5′→3′)	Reference
RsTz2-092-190	RsTz2-092 clade	GCCTTTCTTGCTACACCA	This study
Deferri-term-661	Insect gut-clade	TATTCCGCATTCCTCTCC	This study
Spiro-36	*Spirochaetales*	CTTAAGACGCGCCGCCAG	[6]
EUB338	Most bacteria	GCTGCCTCCCGTAGGAGT	[7]

* Except for RsTz2-092 and its close relatives.

Table S3. Repeat and spacer sequences of CRISPR in the CpT32-4 genome.

	Sequence (5′→3′)	Position
Repeat	CGCACCTTTCACGGGTGCGTGGATTGAAAT	
Spacer 1	ACCGATTGGCAAAAGCTACAACTGCCCGCAGTGC	144,837–144,870
Spacer 2	ACGTAAAATCGAGCCTTTATCCGATACGAGCATGA	144,901–144935
Spacer 3	ACCGCTGGCGGACACGCTATCACAGAGCCGTACATT	144,966–145,001
Spacer 4	ATAGATTTAGGTGTAGTTATAAAGCCATACACTAG	145,032–145,066
Spacer 5	ATTTTCGCACAATGATCATTTACAGCCTCGACAGG	145,097–145,131
Spacer 6	TGTAGTAAAGTGTTAGCAGTTTACTAACAGAGGT	145,162–145,195
Spacer 7	TATTGAGCATGAGCCACTAATCCCGACCCTTATA	145,226–145,259
Spacer 8	AGCTTATTGGATCAAAAAAAGACCGTGTTGAAA	145,290–145,322
Spacer 9	ACAAAGGCCAATAGGCGAGCACAAGTGTGCAAGAG	145,353–145,387
Spacer 10	GTATAGGCAGTGATCGTTACCTAGCTGCAACTCCAG	145,418–145,453
Spacer 11	TGGCTAATCGTAAAATATACGATGCTGACAAACT	145,484–145,517
Spacer 12	TCAAATTATGTGCCGATCATGACACATAAAAAGT	145,548–145,581
Spacer 13	TTATTTTATACTCCATCTCTGCCAGCCGCCGCTT	145,612–145,645
Spacer 14	ATATTGTTACTTTTAGTGCTAACAAACACACCAA	145,674–145,709
Spacer 15	TGAATAGGTAGAAAGGGTGTGTATTATGGAAATG	145,740–145,773
Spacer 16	TCAGCTCTGCGCCATCAACGCCCAGCTCTTTCAG	145,804–145,837
Spacer 17	TATTAATTTATCCTTCCTTATTTTTATGCTTCGG	145,868–145,901

References to Supplementary Materials

1. Takahashi S, Tomita J, Nishioka K *et al.* Development of a prokaryotic universal primer for simultaneous analysis of bacteria and archaea using next-generation sequencing. *PLOS ONE* 2014;**9**:e105592. https://doi.org/10.1371/journal.pone.0105592
2. Hongoh Y, Sato T, Dolan MF *et al.* The motility symbiont of the termite gut flagellate *Caduceia versatili*s is a member of the "*Synergistes*" group. *Appl Environ Microbiol* 2007;**73**:6270–6. https://doi.org/10.1128/AEM.00750-07
3. Taerum SJ, Jasso-Selles DE, Wilson M *et al.* Molecular identity of *Holomastigotes* (Spirotrichonymphea, Parabasalia) with descriptions of *Holomastigotes flavipes* n. sp. and *Holomastigotes tibialis* n. sp. *J Eukaryot Microbiol* 2019;**66**:882–91. https://doi.org/10.1111/jeu.12739
4. Gile GH, James ER, Scheffrahn RH *et al*. Molecular and morphological analysis of the family Calonymphidae with a description of *Calonympha chia* sp. nov., *Snyderella kirbyi* sp. nov., *Snyderella swezyae* sp. nov. and *Snyderella yamini* sp. nov. *Int J Syst Evol Microbiol* 2011;**61**:2547–58. https://doi.org/10.1099/ijs.0.028480-0
5. Sato T, Kuwahara H, Fujita K *et al.* Intranuclear verrucomicrobial symbionts and evidence of lateral gene transfer to the host protist in the termite gut. *ISME J* 2014;**8**:1008–19. https://doi.org/10.1038/ismej.2013.222
6. Hongoh Y, Deevong P, Hattori S *et al.* Phylogenetic diversity, localization, and cell morphologies of members of the candidate phylum TG3 and a subphylum in the phylum Fibrobacteres, recently discovered bacterial groups dominant in termite guts. *Appl Environ Microbiol* 2006;**72**:6780–8. https://doi.org/10.1128/AEM.00891-06
7. Amann RI, Binder BJ, Olson RJ *et al.* Combination of 16S rRNA-targeted oligonucleotide probes with flow cytometry for analyzing mixed microbial populations. *Appl Environ Microbiol* 1990;**56**:1919–25. https://doi.org/10.1128/aem.56.6.1919-1925.1990
